# Fecal High-Mobility Group Box 1 as a Marker of Early Stage of Necrotizing Enterocolitis in Preterm Neonates

**DOI:** 10.3389/fped.2021.672131

**Published:** 2021-06-10

**Authors:** Roberta Vitali, Gianluca Terrin, Francesca Palone, Ilaria Laudadio, Salvatore Cucchiara, Giovanni Boscarino, Maria Di Chiara, Laura Stronati

**Affiliations:** ^1^Division of Health Protection Technologies, Territorial and Production Systems Sustainability Department, Agenzia nazionale per le nuove tecnologie, l'energia e lo sviluppo economico sostenibile (ENEA), Rome, Italy; ^2^Department of Maternal and Child Health, University of Roma La Sapienza, Rome, Italy; ^3^Department of Molecular Medicine, Sapienza University of Rome, Rome, Italy

**Keywords:** feeding intolerance, HMGB1, necrotizing enterocolitis, newborns, preterm

## Abstract

**Introduction:** An early diagnosis of necrotizing enterocolitis (NEC), a major gastrointestinal emergency in preterm newborns, is crucial to improve diagnostic approach and prognosis. We evaluated whether fecal high-mobility group box protein 1 (HMGB1) may early identify preterms at risk of developing NEC.

**Materials and Methods:** A case-control study including neonates admitted at the Neonatal Intensive Care Unit (NICU) of the Sapienza University Hospital “Umberto I” in Rome, from July 2015 to December 2016. Stool samples obtained from cases (preterm newborns with NEC) and controls (newborns without NEC) were collected at the enrolment (T0) and within 7–14 days after the first sample collection (T1). HMGB1, extracted and measured with western blot, was reported as densitometry units (DUS).

**Results:** HMGB1 levels in 30 cases (*n* = 28—Bell stage 1, *n* = 2 Bell stage 2) were higher [T0: 21,462 DUS (95% CI, 16,370–26,553 DUS)—T1: 17,533 DUS (95% CI, 13,052–22,014 DUS)] than in 30 preterm controls [T0: 9,446 DUS (95% CI, 6,147–12,746 DUS)—T1: 9,261 DUS (95% CI, 5,126–13,396 DUS), *p* < 0.001). Preterm newborns showed significant higher levels of HMGB1 (15,690 DUS (95% CI, 11,929–19,451 DUS)] in comparison with 30 full-term neonates with birth weight >2,500 g [6,599 DUS (95% CI, 3,141–10,058 DUS), *p* = 0.003]. Multivariate analysis showed that the risk of NEC was significantly (*p* = 0.012) related to the HMGB1 fecal levels at T0.

**Conclusions:** We suggest fecal HMGB1 as a reliable marker of early NEC in preterm neonates. This study supports further investigation on the role of fecal HMGB1 assessment in managing preterm newborns at risk of NEC. Further studies are advocated to evaluate diagnostic accuracy of this marker in more severe forms of the disease.

## Introduction

Despite significant improvements have occurred in neonatal intensive care, the administration of enteral feedings in preterm infants is a challenging step (1). Whenever possible, enteral nutrition (EN) should always be the preferred method in neonatal feeding. In cases of inability of receiving EN, defined as feeding intolerance (FI), parenteral nutrition (PN) represents the unique nutritional support (2). However, prolonged use of PN is associated with systemic and metabolic complications (3). Thus, EN should be attempted in each preterm neonate since the first day of life (4).

In most cases, FI is a benign condition related to gut function immaturity; however, its presentation may overlap with that of an impending necrotizing enterocolitis (NEC) (5–10). The interpretation of early signs of NEC is still an undefined issue in preterm nutritional care, thus biomarkers helpful in identifying newborns at risk of developing NEC are warranted (5, 8, 11–14). Currently, no biomarker has sufficient predictive value for clinical purposes (14, 15).

It has been suggested that NEC may depend on immaturity of epithelial barrier and innate immunity response to the extrauterine environment (16–18): these components achieve the primary host defense by recognizing microorganisms through pathogen-associated molecular patterns (PAMPs) and by reacting to tissue damage signals, known as damage-associated molecular patterns (DAMPs) (19). We focused on high-mobility group box protein 1 (HMGB1), a DAMP prototype, released from injured immune cells during inflammation (20). HMGB1 is a polypeptide of 215 amino acids in length, mediating inflammation by activating innate immunity through signal transduction in Toll-like receptors (TLRs) and the receptors for advanced glycation end products (21, 22). Increased levels of HMGB1 in the stools have been related to intestinal inflammation in animal models (23). We have previously shown in patients with inflammatory bowel disease (IBD) that fecal HMGB1 is a robust non-invasive biomarker of mucosal inflammation and healing, suggesting its potential role in the diagnostic approach and monitoring intestinal inflammation in children (24, 25).

Thus, we aimed to assess whether fecal HMGB1 may early identify preterms at risk of developing NEC.

## Materials and Methods

### Population and Sampling

This is a prospective case-control study including all preterm neonates consecutively admitted to the Neonatal Intensive Care Unit (NICU) of the Sapienza University Hospital “Umberto I” in Rome, from July 2015 to December 2016. Neonatologists unaware of the study aims (G.B. and M.D.C.) considered eligible for the study newborns when they fulfilled the following criteria: (1) gestational age (GA) between 25 and 36 weeks; (2) body birth weight (BW) between 500 and 2,500 g; (3) no antibiotic treatment before enrolment; (4) parental consent obtained. Newborns with at least one of the following conditions were excluded: (1) Apgar score <5 at 5 min; (2) critical clinical conditions (pH <6.8 on cord blood, or hypoxia with persistent bradycardia for at least 1 h); (3) incomplete clinical data or deviation from nutritional study protocol; (4) maternal history of immunologic, inflammatory, or infectious diseases; (5) surgery; (6) major congenital malformations or inborn errors of metabolism; (7) neonatal congenital infections; and (8) hemodynamic treatment or sedation/analgesia before enrolment.

Among eligible subjects, we enrolled as cases newborns with signs and symptoms suggestive of NEC including biliary or bloody gastric residual, bloody stools, or feeding intolerance (defined by gastric residuals >50% of daily prescribed EN) associated with systemic symptoms (i.e., poor perfusion, muscle hypotonia or hypertonia, lethargy, progressive increase in O_2_ requirement, bradycardia, unstable body temperature, unexplained and persistent metabolic acidosis, unexplained and persistent hyperglycemia) and/or with radiological sign of NEC (10, 26). All newborns without signs and symptoms of NEC during hospital stay served as potential Controls. Each case was matched for GA (±1 week) with one control. Staging of NEC were established according to Bell Stage criteria and confirmed after an agreement between three researchers (G.T., G.B., and M.D.C.) (27). For comparison, we also enrolled healthy babies born at term of gestation or with BW >2,500 g as not-matched controls' group. Two researchers (G.T. and G.B.) confirmed patients' classification in case and in control groups.

We collected fecal samples of all eligible newborns every 72 h and from 3 to 28 days of life. We collected additional fecal samples in the cases, when signs and symptoms suggestive of NEC appeared (T0) and after 7–14 days (T1). All fecal samples were placed into sterile and codified tubes and stored at −80°C. We analyzed fecal samples collected at T0 and T1 of all enrolled cases. Among fecal samples obtained from controls, we selected those useful for the analysis according with postnatal age. In other words, in the control group, we considered T0 the fecal sample collected in the days of life similar (±3 days) with T0 of the matched case and as T1 the fecal sample collected from the same control subject after 7–14 days.

In non-matched controls' group, we collected one fecal sample within the 4th day of life, after first meconium emission. At each time point, the first available sample of at least 0.5 g of formed stool was collected.

### Laboratory Procedures

Researchers (R.V., F.P., I.L., and L.S.) unaware of the clinical data performed laboratory investigations. Stored stool specimens were weighted and resuspended in a volume of extraction buffer (ScheBo Biotech AG, Giessen, Germany) to obtain a final concentration of 500 mg/ml. Samples were placed to a vigorous orbital shaking for 1 h at room temperature and then centrifuged twice for 10 min at 10,000 rpm at 4°C (24, 25). Clear supernatants (fecal extracts) were collected and stored at −80°C. Total protein concentration was determined by the Bradford assay (Bio-Rad Laboratories, Hercules, CA). Twenty micrograms of fecal extracts were fractionated by sodium dodecyl sulfate-(12%) polyacrylamide gel electrophoresis to detect HMGB1. Proteins were transferred in polyvinylidene fluoride membrane (Bio-Rad Laboratories) and blocked with TBS-Tween 20 0.1%, containing 5% nonfat dry milk. Antihuman HMGB1 antibody (1:1,000; R&D, Minneapolis, Minnesota) was diluted in TBS-Tween 20 0.1%, containing 3% nonfat dry milk and incubated overnight at 4°C. Membranes were washed in TBS-Tween 20 0.1%, incubated for 1 h with horseradish peroxidase-conjugated secondary antibody (Santa Cruz Biotechnology Inc., Santa Cruz, California), washed in TBS-Tween 0.1% and developed with LiteBlot EXTEND (Euroclone, Milan, Italy). Densitometric analysis of the western blot bands were performed using the Software ImageQuant Las500 (GE Healthcare Life Science, Uppsala, Sweden). Fecal concentration of fecal HMGB1 was defined on the basis of densitometry scale and expressed as densitometry unit (DUS) (24, 25).

### Data Collection

Investigators not involved in the eligibility and enrolment phases, prospectively recorded prenatal, perinatal, and postnatal data using a structured data form, from birth to discharge, transfer to another hospital, or death. Modalities of EN administration and feeding tolerance were prospectively recorded. Data were reported in a specific coded data form for each enrolled patient. Main neonatal morbidities (moderate–severe BPD, IVH stage ≥2, PLV any stage and early and late-onset sepsis) were diagnosed according to the standard criteria (28–30), by physicians unaware of the study design and aims.

### Feeding Protocol

Mother milk and preterm formula represented the two available options for EN. This was applied as previously described (31), with a minimal enteral feeding (MEF) (10–20 ml/kg/day divided in four to eight feeds) commenced as soon as the general clinical condition was stable. Between 48 and 96 h, our protocol recommends increasing the feeds of 15–30 ml/kg/day according to the BW. Donor human milk was not available, in our NICU, during the study period. Until full enteral feeding was reached, PN was administered through a central vascular access as previously described (32).

### Management of NEC

A supportive medical management of NEC was started promptly, as soon as NEC was suspected. Medical management for suspected and confirmed NEC overlap (33). Pediatric surgical consultation is advised in every case of suspected NEC. Infants with suspected or confirmed NEC were placed nil per os to allow for bowel rest (33). A gastric tube for bowel decompression and monitoring or aspirate were placed. An initial radiograph of the abdomen and left lateral decubitus of cross-table view were obtained to roil out evidenced of free air. Serial and positional abdominal radiograph with a frequency of 1–2/day consistent with the suspicion and cadence of advancing clinical disease, followed the initial series. A complete blood count, including differential in platens counts, electrolyte measurement, blood gas, lactate and indices of liver function and coagulation was performed at least one per day, according with evolution of clinical condition, every 24 h (33). Correction of anemia, thrombocytopenia, electrolyte disturbance, and coagulopathy were performed when necessary. Antimicrobial coverage, broadly targeted gram negative and anaerobic bacteria, where performed for 7–14 days based on clinical suspicion, confirmation disease, and infants' clinical course (33). Infants the developed of NEC stage more than III were an absolution indication for surgical consultation and intervention.

### Ethics

The study was conducted in conformity with World Medical Association Declaration of Helsinki for medical research involving human subjects and after approval by the Ethics Committee of the University Hospital Umberto I in Rome (number 91613). We collected anonymized data after a written informed consent obtained from the parents of each enrolled infant.

### Statistical Analysis

We checked for normality using Shapiro-Wilk test. The mean and standard deviation or median and interquartile range summarized continuous variables and number with percentage summarized categorial variables. We used χ^2^ and exact test for categorical variable, *t*-test, Mann-Whitney test, and Wilcoxon test for paired and unpaired variables. Correlation was assessed with categorical variables by Wilcoxon rank sum tests and with continuous variables by Pearson correlation, between HMGB1 fecal levels, GA and BW. Additional correlations were performed in preterm between HMGB1 fecal levels and days of full enteral feeding (FEF), g/kg of breast milk of the first 7 and 14 days of life. Binary logistic regression analysis was performed using as dependent variables the presence of NEC and as independent variables GA (≤30 or >30 weeks), sex (male or female), type of delivery (cesarean section or vaginal delivery), pH on cord blood (≤7.25 or >7.25), intrapartum antibiotic prophylaxis for prelabor rupture of membranes (not or yes), complete antenatal steroids prophylaxis (not or yes), and value of HMGB1 in the first 7 days of life. The level of significance for all statistical tests was two sides (*p* < 0.05). For statistical analysis, we used Statistical Package for Social Science Software for Microsoft Windows (Chicago, IL, version 25.0) and StatsDirect 3 (Merseyside, UK, version 3.2.10).

## Results

Among a Cohort of 118 premature neonates, 15 were excluded because of maternal history of inflammatory diseases (5), congenital malformations (1), or transfer to other hospital (9). We observed 30 cases and, based on pairing criteria, we selected 30 matched controls. We also enrolled 30 not-matched controls' neonate (born at term of gestation or with BW >2,500 g). Fourteen fecal sampling among cases and controls, and two samples among neonates at term were not analyzed because of inadequate storage or incomplete data collection.

The main clinical characteristics of participating cases and controls were summarized in Table 1. The duration of PN and amount of EN were markedly longer and lower in NEC cases, respectively, than in controls.

**Table 1:** Clinical characteristics of the study population.

	**Cases (*n* = 30)**	**Controls (*n* = 30)**	***p***
Gestational age (weeks)	31.8 (30.8–32.7)	32.5 (31.6–33.3)	0.133
IUGR [*N* (%)]	2 (6.7)	0 (0)	0.150
Intrapartum antibiotic prophylaxis for PROM [*N* (%)]	5 (16.7)	5 (16.7)	1.000
Antenatal steroids^a^ [*N* (%))	20 (66.7)	16 (53.3)	0.292
Cesarean section [*N* (%))	25 (83.3)	19 (66.3)	0.080
Female sex [*N* (%))	9 (30)	18 (60)	0.020
Apgar score at 5 min	8.1 (7.7–8.5)	8.3 (7.9–8.7)	0.406
pH on cord blood	7.28 (7.24–7.31)	7.29 (7.26–7.32)	0.446
Start of EN (DOL)	2.2 (1.3–3.0)	1.4 (0.7–2.0)	0.153
Duration of PN (DOL)	9.6 (6.6–12.7)	4.9 (1.7–8.2)	0.034
EN from 0 to 7 DOL (ml/kg/week)	218 (126–311)	447 (343–552)	<0.001
Breast milk from 0 to 7 DOL (ml/kg/week)	49 (18–80)	145 (75–215)	0.008
Time of the enrollment (DOL)	6.4 (4.2–8.7)	7.2 (3.9–10.6)	0.657
Time of 2nd fecal sample (DOL)	16.5 (13.2–19.8)	16.6 (10.3–22.9)	0.963
BPD [*N* (%)]	1 (3.3)	0 (0)	0.313
IVH [*N* (%))	1 (3.3)	0 (0)	0.313
PVL [*N* (%))	1 (3.3)	0 (0)	0.313
Early-onset sepsis [*N* (%))	1 (3.3)	0 (0)	0.313
Late-onset sepsis [*N* (%))	2 (6.7)	0 (0)	0.150

a *Intramuscular steroid cycle in two doses of 12 mg over a 24-h period. IUGR, intrauterine growth retardation; PROM, prelabor rupture of membranes; EN, enteral nutrition; PN, parenteral nutrition; DOL, days of life; BPD, bronchopulmonary dysplasia; IVH, intraventricular hemorrhage; PVL, periventricular leukomalacia. Data were expressed as mean (95% confidence intervals) when not specified*.

**Figure 1:**
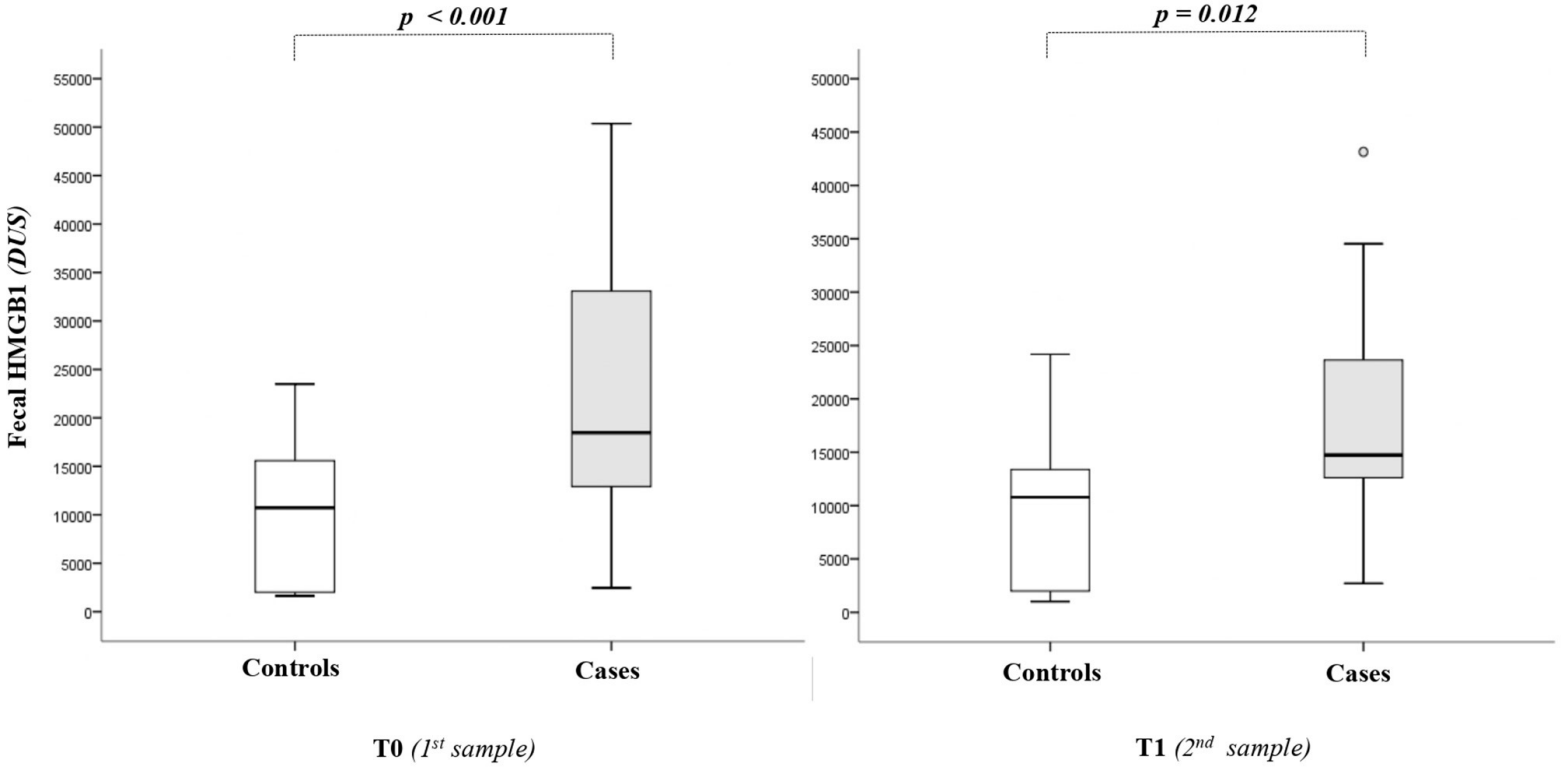
High-mobility group box 1 (HMGB1) fecal levels. The spacing between different parts of the box indicates interquartile range. Horizontal bold black line indicates median. The whiskers above and below the box show the locations of the minimum and the maximum. HMGB1, high-mobility group box 1; DUS, densitometry units; NEC, necrotizing enterocolitis.

**Figure 2:**
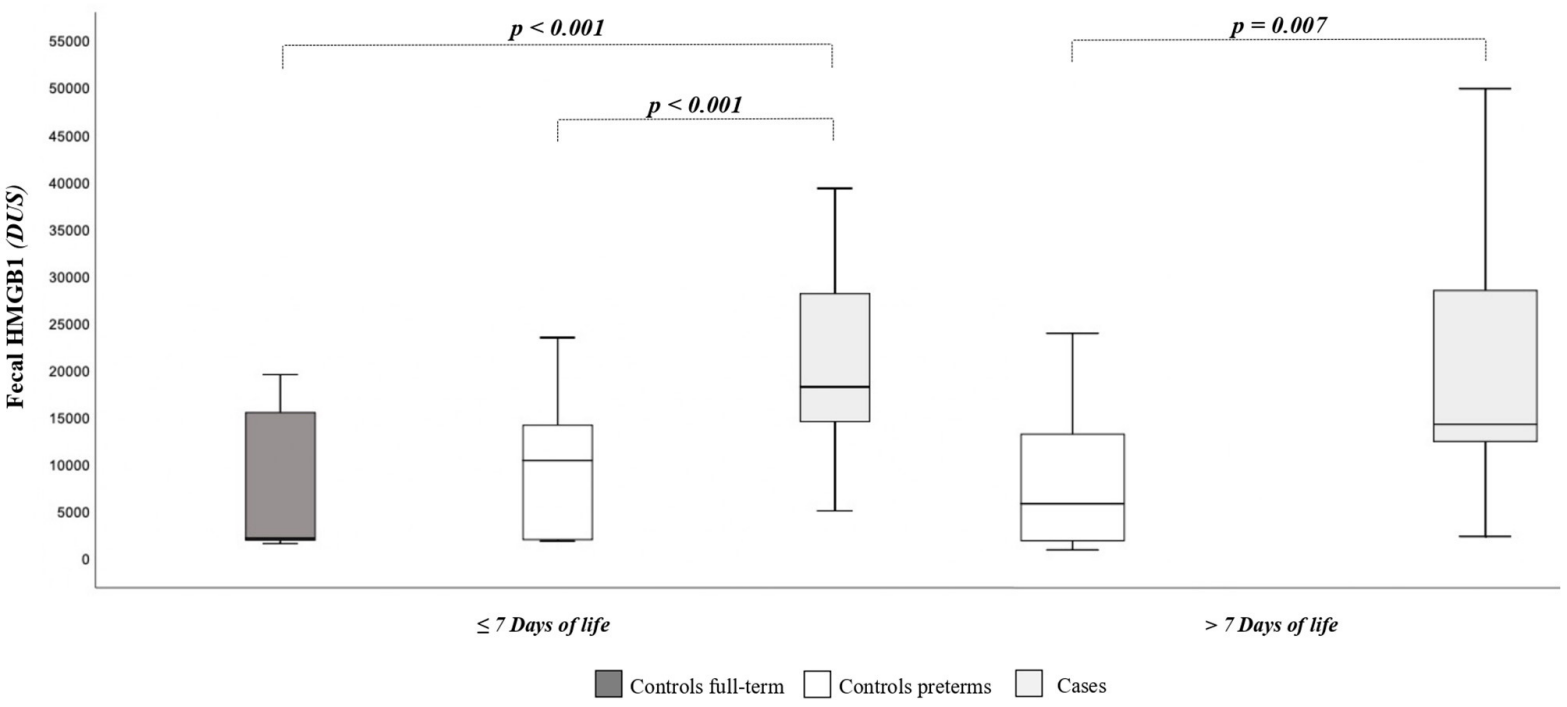
High-mobility group box 1 fecal (HMGB1) levels according to days of postnatal life. The spacing between different parts of the box indicates interquartile range. Horizontal bold black line indicates median. The whiskers above and below the box show the locations of the minimum and the maximum. HMGB1, high-mobility group box 1; DUS, densitometry units; NEC, necrotizing enterocolitis.

As shown in Figure 1, the HMGB1 fecal levels at T0 and T1 were significantly higher in cases than in controls. When HMGB1 values were analyzed according to the age of postnatal life, significant different levels of the marker between cases and controls were detected (Figure 2).

Fecal HMGB1 values in the first week of life in newborns at term of gestation or with BW >2,500 g [GA, 38 weeks (95% CI, 37–39 weeks); BW, 3,194 g (95% CI, 2,954–3,434 g)] were significantly lower than in preterm newborns or with BW <2,500 g [GA, 32 weeks (95% CI, 31–33 weeks); BW, 1,585 g (95% CI, 1,458–1,712 g)]: 6,599 DUS (95% CI, 3,141–10,058 DUS) vs. 15,690 DUS (95% CI, 11,929–19,451 DUS), *p* = 0.003. The HMGB1 fecal levels were significantly correlated with GA (*r* = −0.300, *p* = 0.007) and with BW (*r* = −0.385, *p* < 0.001). The HMGB1 fecal levels at T0 and T1 were not correlated with grams per kilogram of breast milk of the first 7 and 14 days of life, considering all newborns and only cases of controls. At regression analysis, the risk of NEC was significantly related (*p* = 0.025) to the fecal levels of HMGB1 at T0 (Table 2).

**Table 2:** Binary regression analysis to evaluate influence of covariates on the risk of necrotizing enterocolitis developing.

	**B**	**SE**	**Wald**	***p*-value**
GA	3.958	2.039	3.768	0.052
Sex	1.769	1.442	1.506	0.220
Type of delivery	0.953	1.403	0.461	0.497
pH on cord blood	−0.200	1.485	0.018	0.893
Intrapartum antibiotic prophylaxis for PROM	−2.341	3.444	0.462	0.497
Antenatal steroids^a^	−0.501	1.615	0.096	0.757
HMGB1 at T0	0.000	0.000	4.994	0.025
Constant	−5.197	4.241	1.501	0.200

a *Intramuscular steroid cycle in two doses of 12 mg over a 24-h period. PROM, prelabor rupture of membranes; HMGB1, high-mobility group box 1; DOL, days of life*.

We observed that two out of 30 newborns with initial stage of NEC, evolved in NEC stage II and subsequently in NEC stage III. In the remaining, 28 out of 30 symptoms disappeared after medical management. The two patients who had received a diagnosis of NEC stage I evolved within few days to the stage III of disease according to Bell criteria. Fecal HMGB1 concentration at the enrollment of these neonates were 39,231 DUS and 33,537 DUS, respectively; however, fecal samples at T1 were not collected because they underwent surgery.

## Discussion

Our data suggest that fecal HMGB1 can be viewed as a reliable marker of early NEC in preterm newborns. We found reduced fecal concentrations of HMGB1 in newborns at term compared with preterm infants, while increased levels of HMGB1 were detected in the early stages of NEC.

The HMGB1, secreted from different types of cells, is a potent proinflammatory mediator when present extracellularly (20, 21). At least 14 HMGB1 receptors have been identified, however, TLR4 seems to be its major binding site (22, 34–37). It is worthy to note that TLR4 seems to have a crucial role in NEC development (38, 39): its activation inhibits enterocyte migration and leads to apoptosis in mice, *via* NFκB pathway, while its inhibition in the intestinal epithelium of mice and cell cultures prevents NEC development, weakening the degree of enterocyte apoptosis (40, 41). Fetuses express elevated levels of TLR4 until the end of the gestation, thus suggesting decreased ability to repair epithelium after injury in preterm babies (42, 43). Moreover, variables such as gut barrier failure, bacterial translocation, intestinal inflammation, and damage occur during NEC development (16–18).

The diagnosis of NEC currently relies on a combination of clinical and radiological features although there are many drawbacks on their use (10, 11). Several biological markers have been proposed to improve the diagnosis of NEC; however, their clinical usefulness has been hampered by the limited evidence available and the overlap with the ranges of values obtained in healthy and diseased subjects (14, 15). It is widely agreed that FI represents an early clinical manifestation in many newborns that will develop NEC, and commonly follows a prenatal or postnatal gut injury inducing inflammation (5, 6): thus, our results support the assessment of fecal HMGB1 in detecting subjects with FI, thus optimizing the approach to the NEC.

Fecal HMGB1 has been described as a reliable marker of intestinal inflammation in adult and pediatric populations (24, 25), while the usefulness of serum HMGB1 has been reported in the diagnosis of neonatal hypoxia-induced organ damage (42); moreover, modulation of HMGB1 seems to reduce inflammation in experimental colitis (23). Our results suggest a promising clinical use of fecal HMGB1 assessment in preterm newborns observed in NICU, i.e., in the management of EN in preterm newborns at risk of developing NEC (22, 43). However, future well-designed trials are necessary to validate this clinical perspective.

If HMGB1 is a player in the inflammatory cascade of the perinatal gut underlying NEC deserves deep investigation. It is of interest that modulation of gut microbiota through probiotics, in animal models as well as in cell cultures, reduces epithelial damage and HMGB1 levels (44). Since HMGB1 interacts with the innate and adaptive immunity, our study may promote an interest on the relationship between the intestinal immunity and gut microbiota in the perinatal age (43, 45), through advanced investigative tools (46).

This study presents some limitations. The physicians evaluating the eligibility were not blinded for newborns' clinical condition. To limit selection and spectrum bias strict inclusion and exclusion criteria were adopted; we considered all the eligible patients and enrolled both cases and controls consecutively. To minimize information bias, clinical data were collected by researchers different from those who measured HMGB1 levels in the stools, unaware of feeding tolerance.

The small sample size is related at least in part to the inclusion criteria adopted in the study. We enrolled preterms with sign and symptoms suggestive of NEC. Among all preterm newborns observed during study period, we enrolled only controls with GA matched with cases. We included newborns with GA between 25 and 36 weeks. It was random that were enrolled small number of newborns with gestational <30 weeks of postmenstrual age (11 of 60). The reduced number of babies with GA <30 weeks at birth limits the generalizability of the results. Further studies, specially designed to evaluate the relation between GA at birth and fecal levels of HMGB1 are advocated. Levels of HMGB1 may be influenced by protocol adopted for EN administration and the use of breast milk. We adopted the same nutritional protocol for both case and control groups thus, we are not able to evaluate the levels of HMGB1 in relation with modalities of EN administration. Despite we did not find relation between HMGB1 and the use of human milk in the first 2 weeks of life, the study was not designed for this purpose. As the diagnosis of FI is mainly based on clinical ground, the risk of misclassification bias was high: in order to improve this aspect, classification of enrolled newborns as case or control was confirmed after an agreement between two researchers (G.T. and G.B.). The number of newborns with severe NEC, enrolled in this pilot study, is not adequate to calculate accuracy of fecal HGMB1. Thus, we are not able to test this marker in more severe forms of NEC. This represents a major limitation of the study and underlines the utility to evaluate the power of fecal levels of HMGB1 in predicting clinical evolution of NEC in further specific studies. On the other hand, these results showed that fecal HMGB1 levels increased even in mild forms, suggesting a role in diagnosis and in medical management of early stages of NEC, including nutritional strategies. Arbitrary definition of the interval between T0 and T1 fecal sample collection may represent a possible bias. We used a western blot bands that required about more than 24 h for the results. The results of our study encourage the development of other techniques (such as ELISA) test that could improve the clinical utility of HMGB1.

In conclusion, our study suggests that fecal HMGB1 can be a marker of early stage of NEC in preterm newborns, promoting research interest on the clinical role of HMGB1 in the nutritional management of preterm newborns. Assessment of fecal HMGB1 may suggest the appropriate timing of EN, such as the advancement of EN in subjects at high risk of NEC. Finally, considering the relation of HMGB1 with the immunity system, its study may help to clarify pathogenesis of NEC, opening new therapeutic perspectives for this severe neonatal disease.

## Data Availability Statement

The raw data supporting the conclusions of this article will be made available by the authors, without undue reservation.

## Ethics Statement

The studies involving human participants were reviewed and approved by University Hospital Umberto I, Rome. Written informed consent to participate in this study was provided by the participants' legal guardian/next of kin.

## Author Contributions

GT, LS, and SC: design of the study, continuous supervision of the study, and periodic discussion of ongoing results. RV, FP, and IL: development of laboratory methods, statistical analysis of results, and quality control. GB and MD: selection of patients, their follow-up, and treatment (together with GT). All authors contributed to the article and approved the submitted version.

## Conflict of Interest

The authors declare that the research was conducted in the absence of any commercial or financial relationships that could be construed as a potential conflict of interest.
